# Elucidating the biosynthetic and regulatory mechanisms of flavonoid-derived bioactive components in *Epimedium sagittatum*

**DOI:** 10.3389/fpls.2015.00689

**Published:** 2015-09-03

**Authors:** Wenjun Huang, Shaohua Zeng, Gong Xiao, Guoyan Wei, Sihong Liao, Jianjun Chen, Wei Sun, Haiyan Lv, Ying Wang

**Affiliations:** ^1^Key Laboratory of Plant Germplasm Enhancement and Specialty Agriculture, Wuhan Botanical Garden, Chinese Academy of SciencesWuhan, China; ^2^Key Laboratory of Plant Resources Conservation and Sustainable Utilization, South China Botanical Garden, Chinese Academy of SciencesGuangzhou, China; ^3^Institute of Chinese Materia Medica, Chinese Academy of Chinese Medical ScienceBeijing, China

**Keywords:** anthocyanin, bHLH, Epimedium, flavonoid, MYB, transcriptional regulation

## Abstract

Herba epimedii (*Epimedium*), a traditional Chinese medicine, has been widely used as a kidney tonic and antirheumatic medicine for thousands of years. In Epimedium, flavonoids have been demonstrated to be the main bioactive components (BCs). However, the molecular biosynthetic and regulatory mechanisms of flavonoid-derived BCs remain obscure. In this study, we isolated 12 structural genes and two putative transcription factors (TFs) in the flavonoid pathway. Phytochemical analysis showed that the total content of four representative BCs (epimedin A, B, C, and icariin) decreased slightly or dramatically in two lines of *Epimedium sagittatum* during leaf development. Transcriptional analysis revealed that two R2R3-MYB TFs (*EsMYBA1* and *EsMYBF1*), together with a bHLH TF (*EsGL3*) and WD40 protein (*EsTTG1*), were supposed to coordinately regulate the anthocyanin and flavonol-derived BCs biosynthesis in leaves. Overexpression of *EsFLS* (flavonol synthase) in tobacco resulted in increased flavonols content and decreased anthocyanins content in flowers. Moreover, *EsMYB12* negatively correlated with the accumulation of the four BCs, and might act as a transcriptional repressor in the flavonoid pathway. Therefore, the anthocyanin pathway may coordinate with the flavonol-derived BCs pathway in Epimedium leaves. A better understanding of the flavonoid biosynthetic and regulatory mechanisms in *E. sagittatum* will facilitate functional characterization, metabolic engineering, and molecular breeding studies of *Epimedium* species.

## Introduction

Herba epimedii, also known as *Horny Goat Weed* or *Yin Yang Huo*, has been safely used as an important traditional Chinese medicine for more than 2000 years and is prepared from *Epimedium* species in the family Berberidaceae (Guo and Xiao, 2003). *Epimedium sagittatum* (Sieb. et Zucc.) Maxim, together with another four *Epimedium* species, *E. brevicornu* Maxim, *E. pubescens* Maxim, *E. wushanense* T. S. Ying, and *E. koreanum* Nakai, have been recorded in the Chinese Pharmacopoeia Commission (2005). Herba epimedii, mainly as a kidney tonic and antirheumatic medicinal herb, has also been used to treat many diseases such as sexual dysfunction, osteoporosis, cardiovascular disease, and tumors (Wu et al., 2003; Ma et al., 2011). In addition, Epimedium plants are also used as groundcovers and ornamental plants, especially in Japan, Europe and America due to their diversity of color and the shape of the flowers and leaves.

Owing to great beneficial effects on human health, a large mass of literature on the phytochemicals and pharmacology of Epimedium have been extensively studied (Wu et al., 2003; Li et al., 2005; Ma et al., 2011). It is well documented that the main bioactive components (BCs) contributing to the various bioactivities in Epimedium plants are prenylated flavonol glycosides, which are end-products of the flavonol branch of the flavonoid biosynthetic pathway. More than 260 compounds have been isolated in the genus *Epimedium*, and among them, prenylated flavonoids are the major constituents (Ma et al., 2011). Icariin, together with epimedii A, B, and C, is used as the representative biomarker for quality evaluation and chemotaxonomy (Wu et al., 2003; Shen et al., 2007; Xie et al., 2010; Ma et al., 2011). These main BCs are phytochemically and pharmacologically well-characterized, but the existing knowledge about the biosynthetic and regulatory mechanisms of these flavonoid-derived BCs in Epimedium is still limited. An EST database of *E. sagittatum* has been developed in our lab and has accelerated the discovery of genes involved in the flavonoid pathway (Zeng et al., 2010) and consequently the key structural genes of anthocyanin biosynthesis have been recently isolated (Zeng et al., 2013a,b).

Flavonoids are a remarkably large group of plant secondary metabolites that are derived from phenylalanine. The flavonoid biosynthetic pathway is one of the most studied pathways of plant secondary metabolites (Koes et al., 2005; Grotewold, 2006). Most of structural gene encoding enzymes involved in this pathway have been isolated and well characterized from several model species such as *Arabidopsis*, petunia, maize, apple, and grape (Holton and Cornish, 1995; Boss et al., 1996; Honda et al., 2002; Lepiniec et al., 2006). The flavonoid biosynthetic genes can be divided into two subsets: the early genes (*CHS*, chalcone synthase; *CHI*, chalcone isomerase; and *F3H*, flavanone 3-hydroxylase) and the late genes (*DFR*, dihydroflavonol 4-reductase; and *LDOX*, leucoanthocyanidin dioxygenase or *ANS*, anthocyanidin synthase) based on the genes regulated by the MYB/bHLH/WD40 (MBW) transcriptional complex (Quattrocchio et al., 1993; Gonzalez et al., 2008). The wealth of knowledge has demonstrated that the MBW complex governs the phenylpropanoid and flavonoid pathways in many species as well as various cell fates in *Arabidopsis* (Koes et al., 2005; Ramsay and Glover, 2005; Hichri et al., 2011). For example, in *Arabidopsis, AtTT2* (MYB), *AtTT8* (bHLH), and *AtTTG1* (WD40) coordinately control proanthocyanidin biosynthesis through regulating the expression of *BANYULS* (*BAN*) (Baudry et al., 2004), while *AtPAP1*/*AtPAP2* (MYB) together with *AtGL3*/*AtEGL3* (bHLH) and *AtTTG1* regulate anthocyanin biosynthesis (Koes et al., 2005; Gonzalez et al., 2008).

To date, the molecular biosynthesis of flavonoid-derived BCs in Epimedium is still completely unknown, although some key anthocyanin-related genes in Epimedium have been recently reported (Zeng et al., 2013a). Previous studies indicated that metabolic flux in the flavonoid biosynthetic pathway is controlled by substrate competition between FLS and DFR in *Arabidopsis* (Gou et al., 2011). However, the relationship between the anthocyanin branch and the flavonol branch in Epimedium is still unclear. Accumulation of BCs in leaves was developmentally regulated, and our data indicated that the season plays a role in BC accumulation (unpublished). The expression profiles of flavonoid genes have not been investigated in response to the developmental stage. Moreover, it has been reported that BC content and composition varies greatly among different species, populations, and even individuals (Li et al., 2005; Xie et al., 2010), suggesting that the biosynthesis of BCs may be controlled by some regulatory genes such as MYB and/or bHLH TFs. A R2R3-MYB TF, *EsMYBA1*, was recently isolated and found to regulate the flavonoid pathway in Epimedium (Huang et al., 2013a). As we know, many abiotic and biotic stresses can induce flavonoid biosynthesis and accumulation in plants, including UV-light, cold temperature, sucrose, hormones, and pathogens. As such, it was also reported that the accumulation of BCs was induced by phytohormones and sucrose treatment, and several MYB and bHLH TFs were suggested to be involved in the regulation of BC accumulation (Zeng et al., 2013a). However, the proposed role of the MBW complex in the regulation of flavonoid-derived BCs and the metabolic flux relationship between the anthocyanin and flavonol branches remain elusive.

In this study, we utilized an EST database of Epimedium and a homology-based cloning method and successfully isolated 14 full-length cDNA clones and three partial sequences of the flavonoid pathway genes, including 12 structural genes and five putative regulatory genes. To explore the correlation between gene expression profiles and BC accumulation patterns, we examined the expression patterns of the flavonoid-related genes in two lines (HN3 and JX3) of *E. sagittatum* which showed different accumulation patterns of the four BCs (icariin, epimedin A, B, and C) during different leaf developmental stages. As the first enzyme of flavonol biosynthesis responsible for the production of flavonoid-derived BCs, EsFLS was functionally characterized by overexpression in transgenic tobacco. In addition to the MBW transcriptional complex regulating the biosynthesis of flavonoid-derived BCs, the coordinate relationship between the anthocyanin and flavonol branches was suggested, which was also supported by the analysis of gene expression profiles, anthocyanin and four BCs accumulation patterns in red and green leaves. Finally, the putative roles of several representative R2R3-MYB TFs in the regulation of the flavonoid pathway of Epimedium were discussed.

## Results

### Isolation of flavonoid biosynthetic and regulatory genes in epimedium

**Figure 1:**
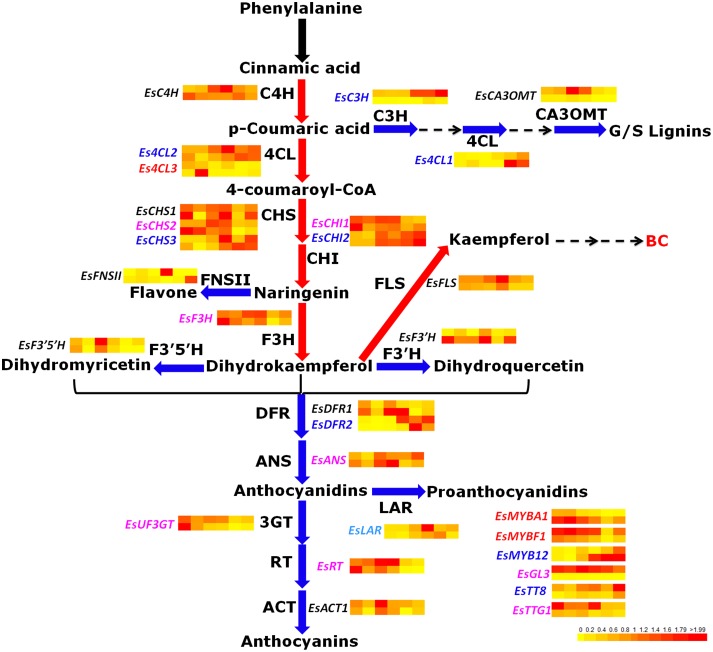
**The predicted flavonoid pathway and the expression patterns of flavonoid-related genes in leaves of ***E. sagittatum*****. Enzyme abbreviations: 4CL, 4-coumarate: CoA ligase; ACT, acyl transferase; ANR, anthocyanidin reductase; ANS, anthocyanidin synthase; C3H, *p*-coumarate 3-hydroxylase; C4H, cinnamate-4-hydroxylase; CA3OMT, caffeic acid 3-*O*-methyltransferase; CHI, chalcone isomerase; CHS, chalcone synthase; DFR, dihydroflavonol 4-reductase; F3′H, flavonoid 3′-hydroxylase; F3′5′H, flavonoid 3′,5′-hydroxylase; F3H, flavanone 3-hydroxylase; FLS, flavonol synthase; FNS II, flavone synthase II; 3GT, 3-glucosyl transferase; LAR, leucoanthocyanidin reductase; RT, rhamnosyl transferase. The blue and red arrows indicate the catalysis steps by enzymes negatively and positively contributing to bioactive component (BC) accumulation in *E. sagittatum*, respectively. The black arrow represents that the first step of the phenylpropanoid pathway catalyzed by the PAL enzyme (phenylalanine ammonia-lyase) is not included for analysis in this study. In both the HN3 and JX3 lines, the genes in blue and red are negatively and positively responsible for BC accumulation, respectively. In either the HN3 or JX3 lines, the genes in light blue and purple are negatively or positively involved in BC biosynthesis, respectively. The genes in black indicate no correlation with BC accumulation. For gene expression, the expression levels of flavonoid-related genes are normalized from 0 to 2 as shown in the lower-right-corner bar and displayed by the BAR HeatMapper Plus Tool (http://bar.utoronto.ca/ntools/cgi-bin/ntools_heatmapper_plus.cgi). For the heat map, the upper-row and bottom-row indicate the expression pattern of genes in the HN3 and JX3 lines, respectively. The six dots in each row correspond to an expression profile of gene obtained at six different developmental stages of leaves.

To date, some key structural genes of anthocyanin biosynthesis were previously isolated from *E. sagittatum*, including *PAL* (phenylalanine ammonia-lyase), *CHS, CHI, F3H, F3*′*H* (flavonoid 3′-hydroxylase), *F3*′*5*′*H* (flavonoid 3′5′-hydroxylase), *FLS, DFR*. and *ANS* (Huang et al., 2012; Zeng et al., 2013a,b). To determine the entire phenylpropanoid/flavonoid biosynthetic pathway, another 12 structural genes encoding enzymes of the flavonoid biosynthetic pathway from *E. sagittatum* were isolated in this study, including the general phenylpropanoid pathway genes (*EsC4H*, cinnamate 4-hydroxylase; *EsC3H, p*-coumarate 3-hydroxylase; and *Es4CL*, 4-coumarate-CoA ligase), the modifying genes encoding enzymes responsible for diverse modification of flavonoids such as acylation, glycosylation, methylation (*EsACT1*, acyltransferase; *EsRT*, rhamnosyltransferase; *EsUF3GT*, UDP-glucose: flavonoid 3-*O*-glucosyltransferase; *EsUF7GT*, UDP-glucose: flavonoid 7-*O*-glucosyltransferase; and *EsCA3OMT*, caffeic acid 3-*O*-methyltransferase) and the branching genes encoding enzymes located at the branching point (*EsFNS II*, flavone synthase II; *EsLAR*, leucoanthocyanidin reductase). Moreover, three copies of *Es4CL* were found in Epimedium, and four putative regulatory genes encoding R2R3-MYB TF (*EsMYBF1*), bHLH TFs (*EsGL3* and *EsTT8*), and WD40-repeat protein (*EsTTG1*) were also isolated (Table S1). Finally, the entire predicted phenylpropanoid/flavonoid biosynthetic pathway in Epimedium was proposed (Figure 1).

**Figure 2:**
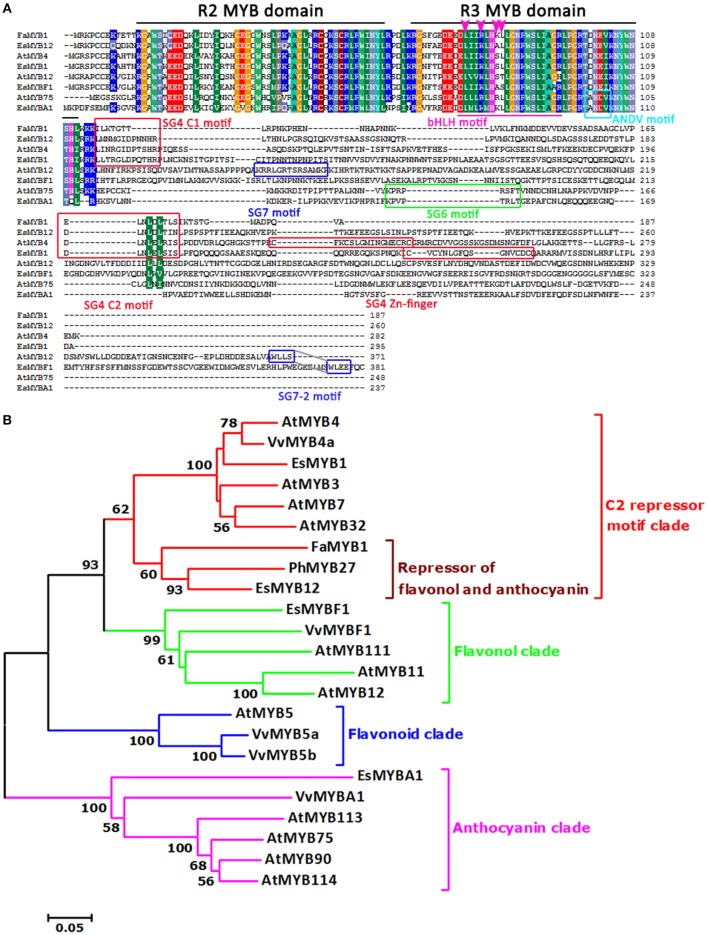
**Multi-alignment and phylogenetic relationship of four R2R3-MYB transcription factors from ***E. sagittatum*** with other plant MYB regulators of the flavonoid pathway. (A)** Alignment of the deduced amino acid sequences of four Epimedium R2R3-MYB proteins and other known plant R2R3-MYB proteins. Identical amino acid residues are shaded in the same color. In addition to the R2 and R3 MYB domains (black lines), the bHLH interaction motif (purple underline) are present in all four EsMYB proteins. The four amino acid residues (LxxRxx[K/R]L) required for the interaction with the maize bHLH protein R is present in EsMYBA1 and marked with an arrowhead. The red boxes represent three conserved motifs, subgroup (SG) 4 C1 (LlsrGIDPx[T/S]HRx[I/L]), SG4 C2 (pdLNL[D/E]Lxi[G/S]) which both are present in the EsMYB1 and EsMYB12 proteins, and SG4 Zn-finger motifs (Cx_1−2_Cx_7−12_Cx_2_C) only present in the EsMYB1 protein. The green and cyan box indicate the SG6 (KPRPR[S/T]F) and ANDV motif, respectively, and both are present in the EsMYBA1 protein. The blue boxes represent the SG7-2 (WLLS) motifs present in the EsMYBF1 and AtMYB12 protein, and the SG7 (KRR[L/p]GRT[G/S]RSAMK) motif present in the AtMYB12 protein. **(B)** The phylogenetic relationship of four Epimedium R2R3-MYB with selected known R2R3-MYB proteins from other plant species using the neighbor-joining method in the MEGA 5 software. The scale bar represents the number of substitutions per site and the numbers next to the nodes are bootstrap values from 1000 replicates, and the values below 50 are not shown. The putative regulatory functions of the R2R3-MYB proteins are indicated, and five distinct clades are formed. All R2R3-MYB protein sequences were retrieved from the GenBank database and accession numbers are as follows: *Epimedium sagittatum*: EsMYB1 (AFH03053), EsMYB12 (AFH03064), and EsMYBA1 (AGT39059); *Arabidopsis thaliana*: AtMYB3 (NP_564176), AtMYB4 (NP_195574), AtMYB7 (NP_179263), AtMYB32 (NP_195225), AtMYB11 (NP_191820), AtMYB12 (O22264), AtMYB111 (NP_199744), AtMYB5 (NP_187963), AtMYB75 (AtPAP1, AAG42001), AtMYB90 (AtPAP2, AAG42002), AtMYB113 (NP_176811), AtMYB114 (NP_176812); *Fragaria x ananassa*: FaMYB1 (AAK84064); *Petunia x hybrida*: PhMYB27; *Vitis vinifera*: VvMYB4a (ABL61515), VvMYBF1 (ACV81697), VvMYB5a (AAS68190), VvMYB5b (AAX51291), and VvMYBA1 (BAD18977).

It was documented that photomorphogenesis may be indirectly associated with the flavonoid pathway. Suppression of the *DET1* gene led to increased carotenoid and flavonoid levels in tomato fruits, as well as phenylpropanoid and anthocyanidins (Davuluri et al., 2005; Enfissi et al., 2010). It is well known that *HY5* plays an important role in photomorphogenic development (Chattopadhyay et al., 1998). The Epimedium homologous genes of the *AtDET1* and *AtHY5* genes were also found in this study (Table S1). For the two R2R3-MYB TFs isolated from Epimedium, *EsMYBA1*, and *EsMYBF1* contained the subgroup (SG) 6 motif and SG7-2 motifs of R2R3-MYB subgroups, respectively. They were clustered into the anthocyanin clade and flavonol clade, which consisted of *MYB* regulators of the anthocyanin and flavonol biosynthetic pathway, respectively (Figure 2), although the conserved SG7 motif was not found in the sequence of *EsMYBF1*. Another two R2R3-MYB TFs identified previously from Epimedium, *EsMYB1* and *EsMYB12*, which are regarded as the putative transcription repressors of the flavonoid pathway (Huang et al., 2013b), were clustered into the C2 repressor motif-containing MYB clade (Figure 2). In particular, *EsMYB12* showed high sequence similarity and a close phylogenetic relationship with strawberry *FaMYB1* and petunia *PhMYB27*, both of which were shown to repress anthocyanin biosynthesis (Aharoni et al., 2001; Albert et al., 2011). As for two bHLH TFs from Epimedium, both *EsTT8* and *EsGL3* contained the helix-loop-helix DNA-binding domain (bHLH) and the MYB-interacting region, and were clustered into two distinct clades that both are involved in the regulation of PAs and anthocyanin biosynthesis (Figure S1). Finally, a total of 32 candidate genes involved in the flavonoid pathway including the structural and regulatory genes shown in Table S2 were investigated further by qPCR assay.

### Accumulation patterns of four bioactive components during the developmental stages of leaves

**Figure 3:**
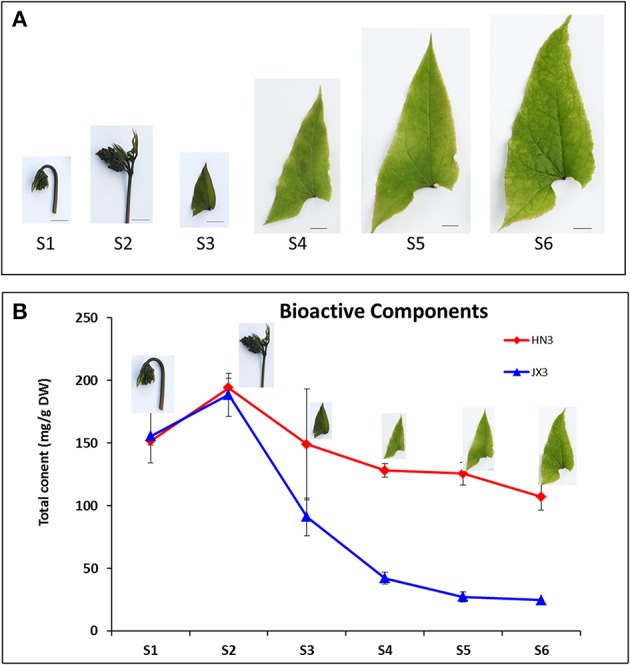
**The developmental stages and the accumulation trend of the total content of four bioactive compounds in leaves of ***E. sagittatum***. (A)** Leaves at six different developmental stages were collected and their phenotypic characterizations are as follows: S1: folded young leaf with hooked petiole; S2: folded young leaf with erected petiole; S3: just expanded young leaf; S4: fully opened young leaf with one-half size mature leaf; S5: no-leathered mature leaf; S6: slightly leathered mature leaf. Bar = 1 cm. **(B)** The total content of four bioactive compounds (icariin, epimedin A, B, and C) in leaves at six different developmental stages from the HN3 and JX3 lines of *E. sagittatum* was measured by HPLC analysis. Data are presented as the mean value plus *SD* (standard deviation) from three biological replicates.

To investigate the accumulation patterns of the four BCs (icariin, epimedin A, B, and C) in Epimedium, leaves at six different developmental stages from S1 to S6 of HN3 and JX3 of *E. sagittatum* were harvested (Figure 3A). HPLC (high performance liquid chromatography) results showed that the accumulation pattern of the total four BCs in HN3 was similar to that of JX3 (Figure 3B). The total content of BCs in both HN3 and JX3 lines reached a peak at stage S2 and then sequentially decreased from stage S3 to S6, in which the total BCs content in HN3 was higher than that in JX3 at each point (Figure 3B). In detail, epimedin C is the major component in both HX3 and JX3 and its accumulation pattern resembled that of the total BCs content in both lines (Figure S2). As for epimedin A, B, and icariin, the contents of those in JX3 were significantly lower than in HN3 (Figure S2). In the HN3 line, icariin peaked at stage S3, while both epimedin A and B peaked at stage S2 (Figure S2). Taken together, the results indicated that epimedin C is the major component of BCs in both lines, and the trend of total content of BCs peaking at stage S2 showed significant divergence as leaves developed, either maintaining a relatively stable level in HN3 or declining remarkably in JX3 during the late developmental stages.

### Correlation analysis of the gene expression profile and the four bioactive components accumulation pattern during leaf development

To investigate the expression profiles of 32 flavonoid-related genes during the developmental stages of leaves, quantitative RT-PCR (qRT-PCR) was carried out, and the correlation between gene expression patterns and four BCs accumulation patterns was analyzed. The expression patterns of flavonoid-related genes were shown in Figure S3, and the correlation analysis results were indicated in the schematic diagram of the flavonoid pathway in Epimedium (Figure 1). In detail, several genes including *EsC3H, Es4CL1, Es4CL2, EsCHS3, EsCHI2, EsDFR2, EsUF7GT, EsMYB12*. and *EsTT8* correlated negatively with the accumulation pattern of the four BCs in both lines, while *Es4CL3, EsMYBF1*. and *EsMYBA1* revealed a positive correlation (Figure 1; Table S3). In addition, seven genes including *EsCHS2, EsCHI1, EsF3H, EsUF3GT, EsRT, EsGL3*. and *EsTTG1* showed a positive correlation with the accumulation of the four BCs in either HN3 or JX3, while the three genes *EsLAR, EsDET1*. and *EsHY5* exhibited a negative correlation (Table S3).

### Coexpression analysis of anthocyanin pathway genes and flavonol pathway genes

**Figure 4:**
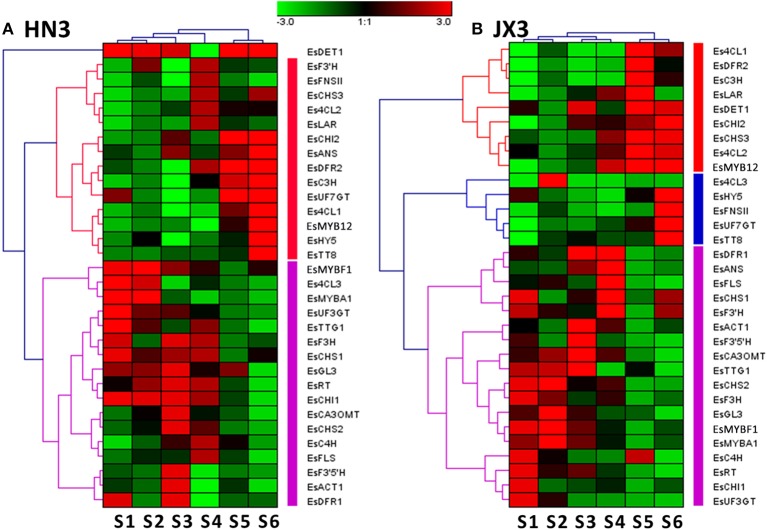
**Hierarchical cluster analysis of gene expression profiles of 32 candidate genes involved in the phenylpropanoid pathway in the HN3 (A) and JX3 (B) lines of ***E. sagittatum*** based on the Genesis method**. Each row represents the relative level of expression for a single gene, and each column shows the expression level for a single sample at a different developmental stage. The intensity of the colors indicates the relative expression level of the gene (see color bar at upper middle).

It is hypothesized that the flavonol branch responsible for BCs production in Epimedium competes with the anthocyanin branch because of the competition of *FLS* and *DFR* genes against the same substrate dihydroflavonol, but whether this competition actually occurs in Epimedium leaves is unknown? A cluster analysis of 32 candidate genes involved in the flavonoid pathway was performed based on the gene expression patterns during leaf development. The results indicated that all the flavonoid-related genes were divided into two large clusters in HN3, one cluster contained the main genes showing a negative correlation with BC accumulation pattern, and the other contained the main genes showing a positive correlation (Figure 4A). In addition, some structural genes of anthocyanin biosynthesis such as *EsCHS2* and *EsDFR1* clustered with *EsFLS*, the first enzyme of the flavonol pathway (Figure 4A). Moreover, the *EsMYBA1* regulator of the anthocyanin pathway also clustered closely with the putative *EsMYBF1* regulator of the flavonol pathway (Figure 4A). For JX3 line, some structural and regulatory genes of the anthocyanin pathway also showed a high similarity of expression patterns with that of the flavonol pathway, as shown by the close clusters of *EsDFR1, EsANS*. and *EsFLS* (Figure 4B). These results suggest that there is likely coexpression between the anthocyanin and flavonol biosynthetic pathways. This supposition was further validated through coexpression analysis. A high correlation was observed between some structural and regulatory genes of both the anthocyanin and flavonol pathways (Figure S4). It is notable that the coexpression was also found among *EsMYBA1, EsMYBF1, EsGL3*. and *EsTTG1*, as they showed a high correlation themselves (Table 1; Figure S4). It is known that the MBW complex regulates the flavonoid pathway in several plant species. Therefore, it is likely that *EsMYBA1, EsMYBF1, EsGL3*. and *EsTTG1* coordinately regulate the biosynthesis of both anthocyanin and flavonol-derived BCs in Epimedium.

**Table 1:** **Correlation analysis of *EsMYBF1, EsMYBA1, EsGL3*. and *EsTTG1* transcription factors from *E. sagittatum* based on the gene expression profiles during the leaf developmental stages**.

**Gene-gene**	**HN3 (R^2^)**	**JX3 (R^2^)**
EsMYBF1-EsMYBA1	0.6239^**^	0.8776^**^
EsMYBF1-EsTTG1	0.4211^**^	0.4427^**^
EsMYBA1-EsTTG1	0.3414^*^	0.3851^**^
EsMYBA1-EsGL3	0.0933^NS^	0.8269^**^
EsGL3-EsMYBF1	0.1648^NS^	0.7069^**^
EsGL3-EsTTG1	0.3264^*^	0.5327^**^

* P ≤ 0.05;

** *P ≤ 0.01; NS, not significant*.

It was demonstrated that MYBs containing the C2-repressor motif act as a transcriptional repressor of the flavonoid pathway. In *Arabidopsis, AtMYB4* represses the transcription of *C4H* to regulate sinapoyl malate accumulation (Jin et al., 2000). It was previously suggested that *EsMYB12* may function as a transcriptional repressor of the flavonoid pathway in Epimedium (Huang et al., 2013b). In this study, it was clear that *EsMYB12* correlated negatively with the accumulation of the four BCs in both lines (Figures S5A,B). Moreover, *EsMYB12* revealed a negative correlation with the *EsMYBA1* and *EsMYBF1* regulators in both lines, as well as *EsGL3* and *EsTTG1* (Figures S4, S5C). In addition, some structural genes of the anthocyanin and flavonol biosynthetic pathways including *Es4CL3, EsUF3GT, EsRT, EsANS, EsFLS*. and *EsF3H* showed a negative correlation with *EsMYB12* to different extents (Figure S5C; Table S4). These results suggested that *EsMYB12* was likely to be a core factor in orchestrating the biosynthetic and regulatory genes of the flavonoid pathway to modulate anthocyanin and flavonol-derived BC biosynthesis. The closer phylogenetic relationship of *EsMYB12* and *FaMYB1* from strawberry also supported this suggestion (Figure 2B), as *FaMYB1* was shown to repress flavonol and anthocyanin accumulation in transgenic tobacco (Aharoni et al., 2001).

### Comparison analysis of gene expression levels and flavonoid accumulation in red and green leaves

**Figure 5:**
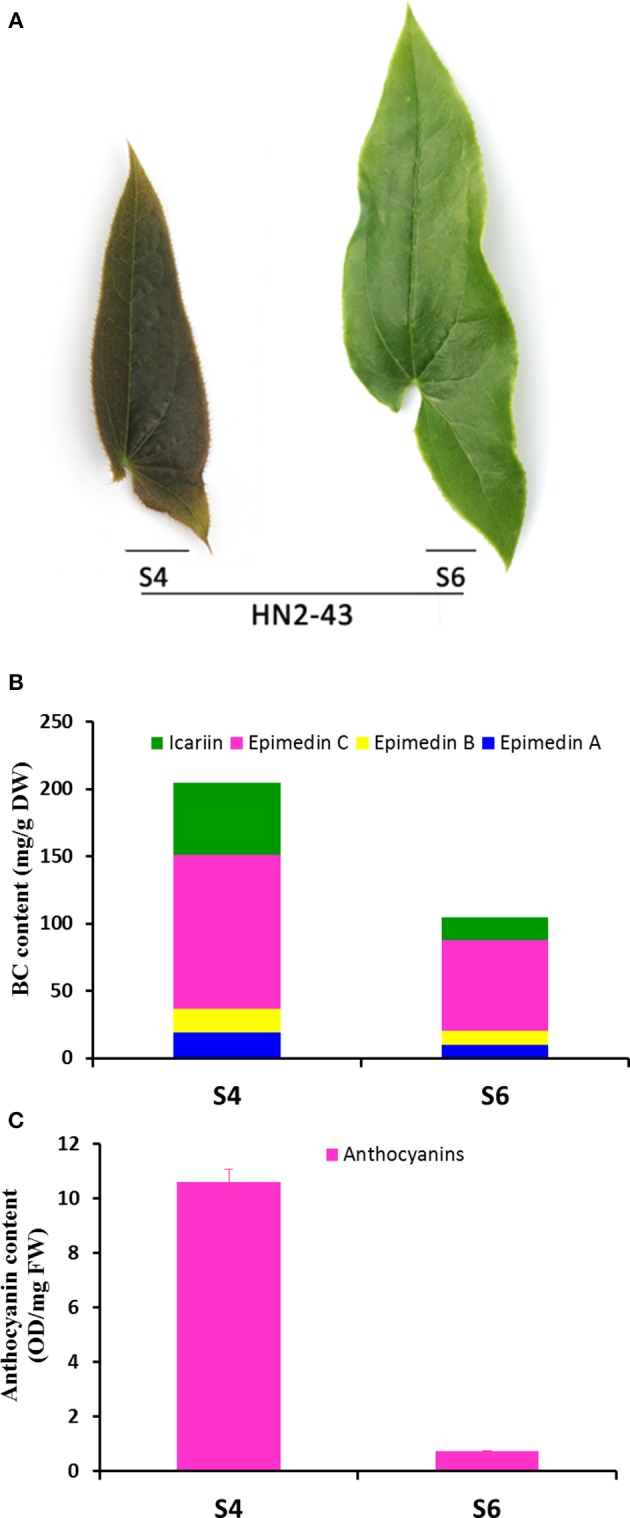
**The phenotype, total content of four bioactive components, and total anthocyanin content in S4 and S6-staged leaves of the HN2-43 line of ***E. sagittatum***. (A)** Leaves of the HN2-43 line show a color change from red (S4) to green (S6) as the leaves mature. Four bioactive components (icariin, epimedin A, B, C) are abundant in the young red S4-staged leaves **(B)** where the total anthocyanin content is also high **(C)** compared to the old green S6-staged leaves.

**Figure 6:**
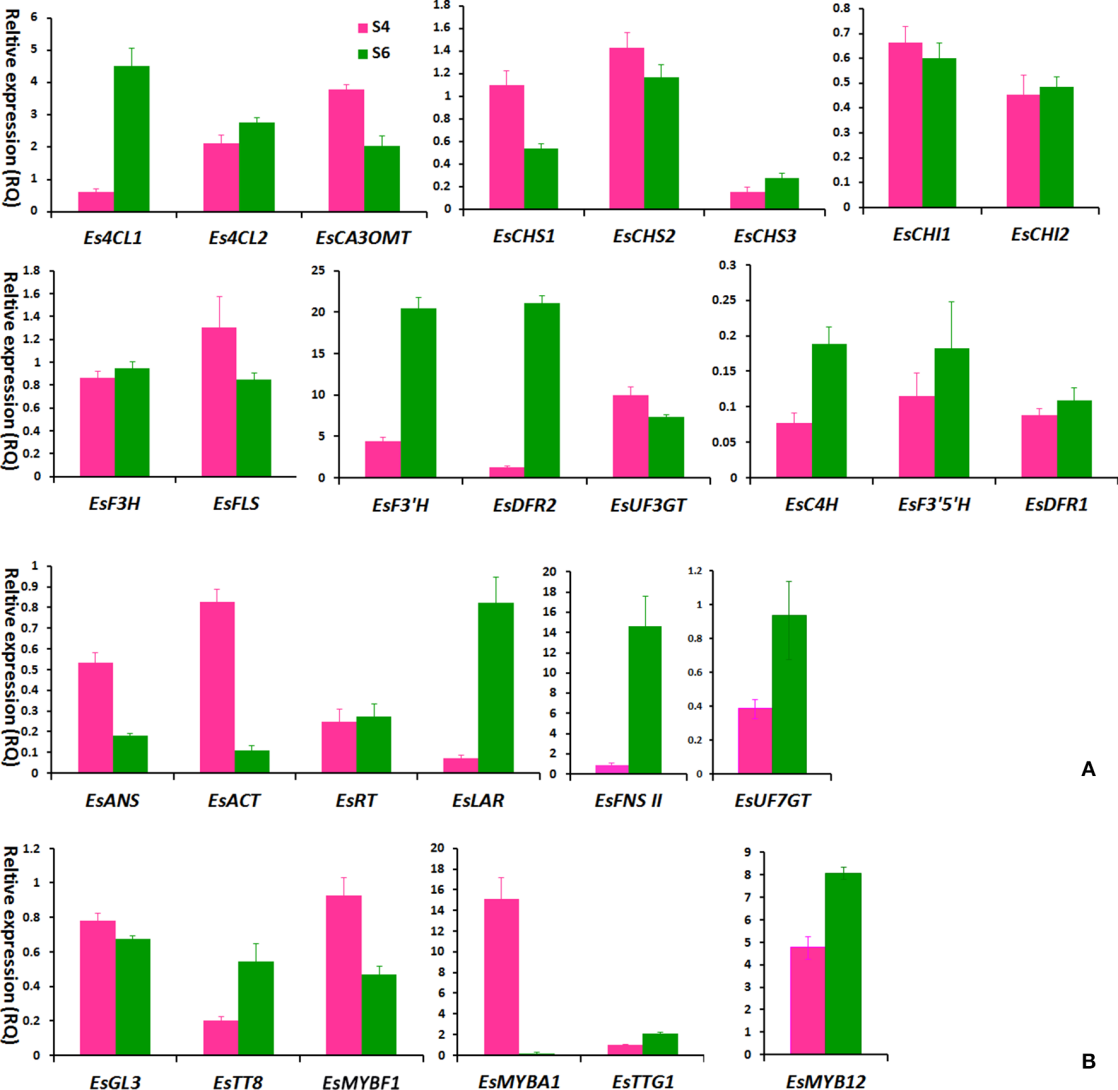
**The expression levels of flavonoid-related genes in S4 and S6-staged leaves of the HN2-43 line of ***E. sagittatum*** by qPCR assay**. Most structural genes **(A)** and several putative regulatory genes **(B)** of the flavonoid pathway are selected for qPCR assay, and each column indicates the mean value plus *SD* (standard deviation) from triple technical replicates. The S4 and S6-staged leaves of HN2-43 are shown in Figure 5.

It was observed that the leaf color of some plantlets of *E. sagittatum* changed from red to green as leaves grew during spring, implying that anthocyanin biosynthesis may be regulated by developmental and environmental factors. To confirm the hypothesis that the anthocyanin pathway coordinates with the flavonol-derived BCs pathway, comparison analysis of the gene expression level and flavonoid accumulation in red and green leaves was carried out. As shown in Figure 5, the young red leaves (HN2-43.S4) contained higher levels of anthocyanin and the four BCs, compared to the old green leaves (HN2-43.S6). The qPCR results showed that some structural and regulatory genes of the anthocyanin pathway were expressed more highly in red leaves than in green leaves, including *EsCHS1, EsCHS2, EsCHI1, EsANS, EsACT1, EsUF3GT, EsMYBA1*. and *EsGL3*, while *EsFLS* and *EsMYBF1* of the flavonol pathway were also expressed more highly in red leaves (Figure 6). In addition, the putative repressor *EsMYB12* was more highly expressed in green leaves than in red leaves (Figure 6B). These results suggested that in Epimedium leaves, the higher the anthocyanin content, the higher the four flavonol-derived BCs content, and the expression patterns of some key biosynthetic genes and several regulatory genes of both the anthocyanin and flavonol pathways correlated with the accumulation pattern of these compounds.

### Overexpression characterization of *EsFLS* in tobacco

**Figure 7:**
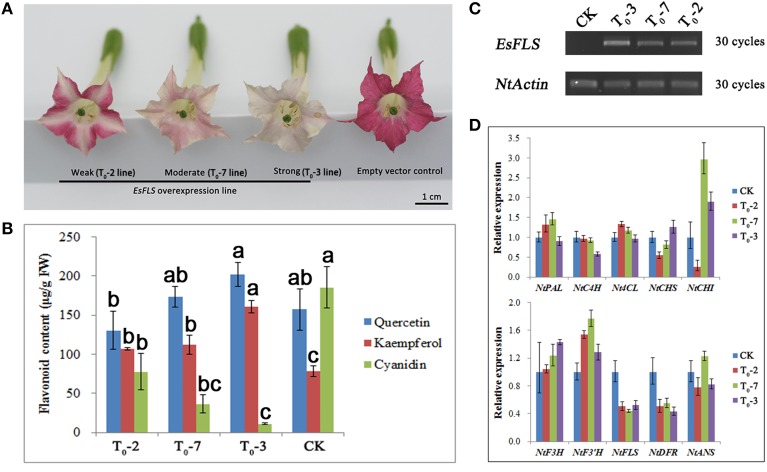
**Overexpression characterization of ***EsFLS*** in transgenic tobacco. (A)** Phenotypic observation of transgenic tobacco overexpressing the *EsFLS* gene and the empty vector pMV as a negative control. **(B)** Measurement of flavonoid content in transgenic tobacco overexpressing *EsFLS* with an empty vector as a negative control (CK). Flavonoid content was determined as aglycones (kaempferol, quercetin, and cyanidin) using HPLC analysis. Each column represents the mean value plus *SD* (standard deviation) from three biological replicates. Columns labeled by the same letter are not statistically significant according to Duncan's multiple range test (*P* ≤ 0.05). **(C)** Detection of *EsFLS* expression in transgenic tobacco by RT-PCR. **(D)** QPCR analysis of the expression levels of flavonoid genes in transgenic tobacco overexpressing *EsFLS* and an empty vector as a negative control (CK). Each column represents the mean value plus *SD* (standard deviation) from three technical replicates.

Due to the important role of the *EsFLS* gene in the main BCs biosynthesis, overexpression of the *EsFLS* gene in tobacco was characterized. It was clearly observed that the flower color of transgenic tobacco plants overexpressing *EsFLS* changed from red to pink, and sometimes even close to white (Figure 7A). HPLC results showed that anthocyanin (cyanidin) decreased and flavonol (kaempferol and quercetin) obviously increased, which corresponded to the extent of color change, compared with the control plants overexpressing the empty vector (Figure 7B). The expression levels of flavonoid pathway genes in transgenic tobacco flowers were measured by qPCR assay. First, it was shown that *EsFLS* was abundantly expressed in the *EsFLS* overexpressing transformants, but was not detected in the empty vector control plant by RT-PCR (Figure 7C). The qPCR results indicated that *NtDFR* was clearly down-regulated and *NtANS* was slightly decreased except for the T_0_-7 transformant compared with the control plant. Some upstream genes of the flavonoid pathway including *NtCHS, NtCHI, NtF3H*, and *NtF3*′*H* increased slightly in the strongly changed T_0_-3 plants than that in the control plants. In addition, the expression levels of three flavonoid biosynthetic genes were not dramatically changed, including *NtPAL, NtC4H*. and *Nt4CL* (Figure 7D). In short, overexpression of *EsFLS* in tobacco resulted in increased flavonols and decreased anthocyanins in flowers, and the metabolic flux of the flavonoid pathway was suggested to be directed into the flavonol branch from the anthocyanin branch based on the results of gene expression levels.

## Discussion

Combined with the previous isolation of some structural genes of anthocyanin biosynthesis, more structural genes of the flavonoid pathway were isolated in this study, leading to elucidation of the entire phenylpropanoid/flavonoid pathway in Epimedium. Moreover, several putative regulators involved in the regulation of the flavonoid pathway were also isolated. Through analyses of gene expression patterns and the accumulation patterns of the four BCs, we found that some genes correlated positively or negatively with the accumulation of the four BCs in leaves of both HN3 and JX3, especially some regulatory genes showing high degrees of correlation such as *EsMYBA1, EsMYBF1*, and *EsMYB12* TFs. This was consistent with the previous report that two R2R3-MYB, *EsMYBF1* and *EsMYB12*, responded to the BC accumulation better than most flavonoid-related structural genes under plant hormone and sucrose treatments (Zeng et al., 2013a). It seemed that most of the branching genes of the flavonoid pathway correlated negatively with the BC accumulation to different extents, including *EsFNS II, EsLAR*. and *EsC3H* (Figure S3; Table S3), which partly directed the main metabolic flux into the flavone, proanthocyanidins (PAs), and lignin biosynthetic branches, respectively. Because two copies of *DFR* exist in Epimedium, *EsDFR2* was suggested to be involved in PA biosynthesis (Zeng et al., 2013a). Corresponding to that, *EsDFR2* showed a negative correlation with BC accumulation (Table S3). In addition, a previous study indicated that photomorphogenesis may be indirectly involved in the regulation of the flavonoid pathway (Chattopadhyay et al., 1998; Davuluri et al., 2005). However, the photomorphogenesis regulatory genes *EsDET1* and *EsHY5* isolated in this study showed a very low negative correlation with the BC accumulation in either HN3 or JX3 (Table S3).

Many MYB TFs have been functionally shown to regulate the flavonoid biosynthetic pathway, and play critical roles in plant development and stress defense. In Epimedium, we found several putative R2R3-MYB TFs involved in the regulation of the flavonoid pathway. In this study, *EsMYBF1* was suggested to be involved in regulating the flavonol biosynthetic pathway, while *EsMYB1* and *EsMYB12* were found to contain the C2-repressor motif which is involved in transcriptional repression. Although *EsMYBF1* showed high sequence similarity with other MYB members of subgroup 7 (SG7) flavonol clade of the R2R3-MYB family, *EsMYBF1* did not correspond well to the target *EsFLS* gene expression during leaf development (Figure S6). This does not seem consistent with the report that *VvFLS1* was the target gene of *VvMYBF1*, a flavonol-specific R2R3-MYB regulator in grape (Czemmel et al., 2009). In *Arabidopsis*, the FLS enzyme is encoded by a family of six genes (Owens et al., 2008), suggesting that a copy of *FLS* correlating with the *EsMYBF1* gene in Epimedium has perhaps not been found. Of the two C2 repressor motif-containing *MYB* genes, only *EsMYB12* correlated negatively with the accumulation pattern of the four BCs during leaf development, suggesting that it perhaps repressed the flavonol-derived BC biosynthesis (Figure S5). Moreover, *EsMYB12* also showed a negative correlation with some structural genes of the flavonoid pathway, even with *EsMYBF1, EsMYBA1*. and *EsGL3* TFs regulators (Figures S4, S5). It is well known that the MBW complex is formed to control the flavonoid biosynthetic pathway in plants, and the MYB repressor (*AtMYBL2* and *AtCPC*) is involved in disturbing the active MBW complex formation for anthocyanin biosynthesis (Matsui et al., 2008; Zhu et al., 2009). Here, it may be possible that *EsMYB12* prevented R2R3-MYB regulators from forming the active complex with bHLH for flavonoid biosynthesis.

It was first hypothesized that the anthocyanin biosynthesis branch may compete with the flavonol biosynthetic branch due to the competition of the *DFR* and *FLS* genes against the same substrate dihydroflavonol (Figure 1). When *EsFLS* was expressed in transgenic tobacco, the endogenous *NtDFR* was down-regulated (Figure 7), suggesting that the main metabolic flux was directed from the anthocyanin to the flavonol branch because of the competition of the introduced *EsFLS* and endogenous *NtDFR*. However, in Epimedium, the gene expression cluster and coexpression analysis suggested that the main structural genes for both anthocyanin and flavonol branch pathways revealed a reliable coordinate relationship rather than competition. In particular, the *EsMYBA1* regulator of anthocyanin biosynthesis coexpressed with the putative *EsMYBF1* regulator of flavonol biosynthesis (Figure 4 and Figure S4), suggesting that these two biosynthetic pathways did not compete. It was observed that the leaves of Epimedium usually showed a red color at the early development in the cold spring, implying that amounts of anthocyanins accumulate in the young leaves. The four flavonol-derived BCs that revealed a high level at the early developmental stage of leaves corresponded to anthocyanin accumulation (Figure 3). The abundant accumulation of both anthocyanin and flavonol-derived BCs in the young leaves of Epimedium in early development might be induced by low temperature in early spring and consequently protect plantlets from biotic and abiotic stresses. It had been reviewed that phenylpropanoid compounds could be induced by various biotic and abiotic stresses, such as cold temperature and UV-light (Dixon and Paiva, 1995). For the plantlets showing a color change from red to green during development, the gene expression analysis and measurement of anthocyanin and four BCs content also support the coordination of anthocyanin and flavonol biosynthetic branches, and the key genes of both pathways corresponded to the accumulation of anthocyanin and the four BCs (Figures 5, 6).

In summary, we have isolated 12 structural genes and five putative regulatory genes of the flavonoid biosynthetic pathway to complete the phenylpropanoid/flavonoid pathway in the medicinal plant *E. sagittatum*. Transgenic tobacco indicated that the *EsFLS* gene is functionally active and conserved. Moreover, two *R2R3-MYB* genes, *EsMYBA1* and *EsMYBF1*, together with *EsGL3* and *EsTTG1* were supposed to coordinately regulate the anthocyanin and flavonol branch pathways in Epimedium leaves, which did not show competition with one another as expected. In addition, the putative *EsMYB12* transcriptional repressor possibly plays a core role in the regulation of flavonol-derived BC biosynthesis via orchestrating other regulatory TFs and biosynthetic genes of the flavonoid pathway. Understanding of the flavonoid pathway in Epimedium therefore provides insight into the molecular biosynthesis and regulation of flavonol-derived BCs, paving the way to the production of the desired products with metabolic engineering. The detailed function of the MBW complex in regulating the flavonoid pathway in Epimedium will be further characterized, particularly the role of *EsMYB12* involved in the imbalance of the MBW transcriptional complex.

## Materials and methods

### Plant materials

Plants of HN3 and JX3 lines of *E. sagittatum*, which were originally isolated from Hunan and Jiangxi province, respectively, were grown in the experimental field of Wuhan Botanical Garden, China. Each sample from the HN3 and JX3 lines was collected at different stages of leaf development and divided into two parts; one was frozen immediately in liquid nitrogen and stored at −70°C for RNA isolation and gene expression analysis, while the other was dried in shade until use for HPLC analysis.

### Isolation of flavonoid-related genes in epimedium

To isolate the potential flavonoid-related genes of Epimedium, an EST database (NCBI accession: SRA008151) of *E. sagittatum* leaves developed in our group (Zeng et al., 2010) was queried using the gene homologs with known functions from other plant species, including *4CL, ACT, C3H, C4H, CA3OMT, FNS II, UFGT, LAR*. and *RT*, and some R2R3-MYB, bHLH TFs and WD40 protein. Moreover, the homology-based cloning method and RACE (rapid amplification of cDNA ends) techniques were also used to isolate the full-length cDNAs of flavonoid-related genes. Furthermore, several fragments homologous to *HY5* and *DET1* genes of *Arabidopsis* were also included for further analysis. Finally, a total of 14 full-length cDNAs of candidate genes involved in the flavonoid pathway of *E. sagittatum* have been successfully isolated, and the partial sequences of several genes as well (Table S1).

### Overexpression vector construct and plant transformation

Overexpression of *EsFLS* gene in tobacco (*Nicotiana tabacum*) was carried out using *Agrobacterium*-mediated leaf disk transformation (Horsch et al., 1985). The full-length cDNA of *EsFLS* was cloned into the modified binary vector pMV (derived from pBI121 vector) to form the pMV-EsFLS construct under the control of the CaMV 35S promoter. This construct pMV-EsFLS was electroporated into the *Agrobacterium tumefaciens* strain EHA105 for tobacco transformation. Transformed plants were selected using kanamycin (100 μg/mL) as a plant selective marker and the presence of exogenous transgene was detected by PCR. A total of nine transgenic tobacco plants overexpressing the *EsFLS* gene was observed to show obvious flower color changes, and three representative transformants were selected for further analysis. In addition, transgenic tobacco plants overexpressing the pMV empty vector were used as the negative control.

### Gene expression profiling by qPCR assay

To detect the expression levels of flavonoid-related genes during the different developmental stages of leaves in Epimedium and in transgenic tobacco flowers at blooming stage, qRT-PCR was carried out as previously described (Huang et al., 2013b). Total RNA was extracted from both Epimedium leaves and tobacco flowers using RNAiso Plus reagent (Takara, Japan). The quality and quantity of the RNA solution were checked by gel electrophoresis and NanoDrop 2000 spectrophotometer (Thermo Scientific, USA). One microgram of total RNA was used for reverse transcription with PrimeScript RT reagent kit, and the accessary gDNA eraser was used to remove any contaminated genomic DNA (Takara, Japan). The qPCR system was recommended by SYBR Premix Ex Taq II kit instructions (Takara, Japan) and run on ABI 7500 Real-Time PCR system (ABI, USA). After the end of qPCR program, melting curve steps were performed to confirm the target product specificity. Gene-specific primers (GSP) for qPCR assay of Epimedium and tobacco were listed in Tables S2, S5, respectively. The *actin* homolog of Epimedium and *tub1* gene of tobacco were used as an internal control. The comparative Ct method was used to determine the relative gene expression levels.

### Phytochemical assay

Leaf tissues of *E. sagittatum* were collected at the different developmental stages and dried in shade, powdered and sieved (40 mesh) for use. Tobacco flowers were harvested at the blooming stage, frozen in liquid nitrogen and stored at -70°C until use. HPLC analysis of four representative BCs (icariin, epimedin A, B, C) in Epimedium was performed according to the previous method (Zhang et al., 2008). Extraction of the four BCs was carried out via an ultrasonic-assisted extraction procedure. In brief, an amount of 50 mg sample powder was immersed in 5 ml 70% ethanol and sonicated for 30 min, and filtered through a 0.22 μm filter membrane (Millipore, USA) prior to injection into the HPLC system. Flavonoid analysis in transgenic tobacco flowers was carried out as described by Vimolmangkang et al. (2013) with minor modification. Anthocyanins and flavonols were extracted from 100–200 mg of finely ground tobacco flowers in 3 ml 1% HCl/methanol (v/v) and 80% methanol, respectively. Anthocyanins were centrifuged after incubation in the dark at 4°C for 24 h, while flavonol extract was sonicated for 30 min, and then kept at 4°C overnight and centrifuged. Flavonoid content in tobacco was determined as aglycones by preparing acid-hydrolyzed extracts. An aliquot of 400 μL of the supernatant was transferred to a fresh tube, acid-hydrolyzed by adding 120 μL of 3 N HCl, incubated at 90°C for 1 h, and then mixed with 200 μL of methanol. The hydrolyzation solution was filtered through a 0.22 μm filter membrane (Millipore, USA) prior to injection. An Agilent 1100 series HPLC system equipped with an Agilent TC-C18 column (5 μm, 4.6 × 250 mm) was used for chromatographic analysis. The HPLC conditions for BC separation in Epimedium were as follows: the mobile phase consisted of solvent A (acetonitrile) and solvent B (1% acetic acid in water, v/v). The gradient elution program was: 0–10 min, 20% solvent A; 10–40 min, 20–25% solvent A; 40–42 min, 25–50% solvent A; 42 min, 50–20% solvent A. Meanwhile, HPLC analysis for flavonoids in tobacco was different, following a previously described method with minor modification (Sun et al., 2013). The mobile phases consisted of solvent A (0.1% formic acid in water) and solvent B (acetonitrile) and solvent C (methanol), and the gradient program for cyanidin, kaempferol, and quercetin was as follows: 0 min, 10% B + 2% C; 10 min, 20% B + 4% C; 15 min, 50% B + 10% C; 20 min, 20% B + 4% C; 25 min, 10% B + 2% C; 28 min, 10% B + 2% C. The column was maintained at 25°C and the solvent flow rate was 1.0 ml/min for both Epimedium and tobacco. The Epimedium and tobacco sample injection volumes were 5 and 10 μL, respectively. The detection wavelength was set at 270 nm for BCs in Epimedium, and at 350 nm for kaempferol and quercetin, at 530 nm for cyanidin in tobacco. The flavonoid compounds were identified with reference to commercial standards of epimedin A/B/C, icariin, cyanidin, kaempferol and quercetin, and their quantification was measured according to the standard curve of each reference.

Total anthocyanin was extracted and measured from green and red leaves of *E. sagittatum* as previously described (Huang et al., 2012). In brief, an amount of 50–100 mg of fresh tissues was powdered and immersed in methanol (containing 1% HCl) in the dark at 4°C overnight with occasional shaking. The extracts were centrifuged at 12,000 g for 10 min and the supernatant was filtered with a 0.22 μm filter membrane and monitored at 530 and 657 nm. The equation A530-0.25 × A657 was used to compensate for the absorption of chlorophyll and its degradation products at 530 nm. Total anthocyanin content was presented as the subtracted absorbance/fresh weight.

### Statistical analysis

Statistical analysis of gene expression and phytochemical data was carried out with the software SPSS version 18.0 (IBM SPSS Statistics). Correlation analysis was performed using Pearson's correlation coefficient (Pearson's *r*) with a two-tailed test. Correlations were considered statistically significant at *P* ≤ 0.05 or *P* ≤ 0.01.

## Genbank accession numbers

The full-length cDNA sequences of flavonoid-related genes isolated in this study have been deposited in the GenBank database, and their accession numbers are as follows: *Es4CL1* (KJ010520), *Es4CL2* (KJ010521), *Es4CL3* (KJ010522), *EsC3H* (KJ010523), *EsC4H* (KJ010524), *EsCA3OMT* (KJ010525), *EsFNS II* (KJ010526), *EsLAR* (KJ010527), *EsACT1* (KJ010528), and *EsGL3* (KJ010529).

### Conflict of interest statement

The authors declare that the research was conducted in the absence of any commercial or financial relationships that could be construed as a potential conflict of interest.

## Supplementary material

Table S1 **Detailed information about the flavonoid-related genes isolated in this study**.

Table S2 **List of primer sequences for the qPCR assay in Epimedium**.

Table S3 **Correlation analysis of flavonoid-related genes regarding the gene expression profiles and the accumulation patterns of the four BCs in the HN3 and JX3 lines**.

Table S4 **Correlation analysis of expression levels between *EsMYB12* and other structural genes involved in the phenylpropanoid pathway**.

Table S5 **List of primer sequences for the qPCR assay in tobacco**.

Figure S1 **Phylogenetic relationship analysis of two bHLH transcription factors, EsTT8 and EsGL3, from *E. sagittatum* and other plant bHLH proteins**. This phylogenetic tree was constructed using the neighbor-joining method by the MEGA 5 software. The scale bar represents the number of substitution per site and the numbers next to the nodes are bootstrap values from 1000 replicates. All bHLH protein sequences were retrieved from the GenBank database and accession numbers are as follows: *Epimedium sagittatum*, EsTT8 (AGT39063); *Petunia x hybrida*, PhAN1 (AAG25927), PhJAF13 (AAC39455); *Lotus japonicus*, LjTT8 (BAH28881); *Arabidopsis thaliana*, AtTT8 (CAC14865), AtGL3 (NP_680372), AtEGL3 (NP_176552); *Zea mays*, ZmIN1 (AAB03841); *Oryza sativa*, OsRc (BAF42668); *Antirrhinum majus*, AmDelila (AAA32663).

Figure S2 **The accumulation patterns of epimedin A, epimedin B, epimedin C, and icariin in the HN3 and JX3 lines of *E. sagittatum* during the leaf developmental stages**. Each data represents the mean value plus *SD* (standard deviation) from three biological replicates.

Figure S3 **The expression profiles of flavonoid-related genes in the HN3 and JX3 lines of *E. sagittatum* during the six developmental stages of leaves**. The comparative Ct method was used to determine the relative expression level. Each date represents the mean value plus *SD* (standard deviation) error from three technical replicates.

Figure S4 **Coexpression analysis of 32 candidate genes involved in the phenylpropanoid/flavonoid pathway in the HN3 (A) and JX3 (B) lines of *E. sagittatum* based on the Genesis method**. The correlation coefficient R is indicated by the color bar at upper right corner.

Figure S5 **The expression profile (A) and correlation analysis of *EsMYB12* in relation to the four BC accumulation patterns (B) with other flavonoid-related genes (C) during the developmental stages of *E. sagittatum* leaves**.

Figure S6 **The expression patterns of the *EsMYBF1* and *EsFLS* genes in the HN3 and JX3 lines of *E. sagittatum* during the developmental stages of leaves**.
