# Supplementary material for: Elucidating the biosynthetic and regulatory mechanisms of flavonoid-derived bioactive components in Epimedium sagittatum
Source: Front Plant Sci. 2015 Sep 3;6:689. doi: 10.3389/fpls.2015.00689 (PMC4558469; doi:10.3389/fpls.2015.00689)
Supplement: Table S1 — Detailed information about the flavonoid-related genes isolated in this study. [file Table1.DOCX]

**Table S1. Detailed information about the flavonoid-related genes isolated in this study.**

| **Genes** | **Subject genes/Accession** | **Species** | **E-value** | **Annotation** |
| --- | --- | --- | --- | --- |
| *Es4CL1* | *Nt4CL1*/O24145 | *Nicotiana tabacum* | 0 | 4-coumarate--CoA ligase |
| *Es4CL2* | *Vv4CL*/AEX32786 | *Vitis vinifera* | 0 | 4-coumarate--CoA ligase |
| *Es4CL3* | *At4CL*-like 4 /NP_173474 | *Arabidopsis thaliana* | 0 | 4-coumarate--CoA ligase-like |
| *EsC3H* | *CYP98A37*/ABC59086 | *Medicago truncatula* | 0 | cytochrome P450 monooxygenase CYP98A37 |
| *EsC4H* | *CYP73A4*/P48522 | *Catharanthus roseus* | 0 | Cinnamic acid 4-hydroxylase, Cytochrome P450 73 (CYP73A) |
| *EsCA3OMT* | *PdCAOMT*/Q43609 | *Prunus dulcis* | 1.00E-129 | Caffeic acid 3-O-methyltransferase |
| *EsFNS II* | *GmFNS II*/ACV65037 | *Glycine max* | 3.00E-155 | flavone synthase II, cytochrome P450 93B |
| *EsLAR* | *FaLAR*/ABH07785 | *Fragaria x ananassa* | 7.00E-145 | leucoanthocyanidin reductase |
| *EsRT* | *PaRT*/ABO21828 | *Petunia axillaris* | 4.00E-81 | UDP rhamnose:anthocyanidin-3-glucoside rhamnosyltransferase |
| *EsUF3GT* | *MtGT*/XP_003593675 | *Medicago truncatula* | 6.00E-161 | Anthocyanidin 3-O-glucosyltransferase |
| *EsUF7GT* | *MdUF7GT*/AAX16493 | *Malus x domestica* | 1.00E-158 | UDP-glucose:flavonoid 7-O-glucosyltransferase |
| *EsACT1* | *VvACT*/XP_002275913 | *Vitis vinifera* | 7.00E-101 | anthocyanin 5-aromatic acyltransferase |
| *EsGL3* | *PhAn1*/AAG25927 | *Petunia x hybrida* | 0 | a bHLH TF regulates the anthocyanin biosynthetic pathway |
| *EsTTG1* | *LjTTG1*/BAH28880 | *Lotus japonicus* | 6.00E-52 | WD40 repeats protein |
| *EsMYBF1* | *VvMYBF1*/ACV81697 | *Vitis vinifera* | 1.00E-23 | a MYB TF regulate flavonol synthesis in developing grape berries |
| *EsDET1* | *AtDET1*/AAB59299 | *Arabidopsis thaliana* | 2.00E-67 | Light-mediated development protein |
| *EsHY5* | *AtHY5-like*/NP_850605 | *Arabidopsis thaliana* | 2.00E-50 | transcription factor HY5-like protein |

**Table S2. List of primer sequences for the qPCR assay in Epimedium.**

| **Genes** | **Forward sequence (5'-3')** | **Reverse sequence (5'-3')** |
| --- | --- | --- |
| *Es4CL1* | ACAAAGGGTTCCAGGTCGCTC | ATCCTTCATCGGGACAACAGC |
| *Es4CL2* | CACAGTAGTCAGGAACGCAGAGC | CGTGGAATCAGCATCATTTAGGT |
| *Es4CL3* | TCACCGATGCTGCTGTTATTCC | ATTCCATGACTGCGGTTTCTGAC |
| *EsC3H* | CAGTGGAATGGGCTATGG | GGAAGGTTGGTGAAATCG |
| *EsC4H* | GACCGTTCCTTTCTTCAC | GCATCTTCCTCACATCTTC |
| *EsCA3OMT* | TAATAGCAAAAGCAGGTGAAG | GCAGTGATGACGGAATAAC |
| *EsCHI1* | CAGAGAAGGTGATAGAGAATTG | GGATGGATGTACCAGGAG |
| *EsCHI2* | AGCATCTTCATCTTCAGTTG | CAGCATACACCTTCACAC |
| *EsCHS1* | GGAAGTCTGAGGAGGAAG | CACATGAACACATACACAATC |
| *EsCHS2* | GTGACGGTGCTATTGATG | AGGCTCTTCTGGATGTTC |
| *EsCHS3* | CTGACTATTACTTCCGCATTAC | CACCTCCACTACTACCATATC |
| *EsF3H* | TCGTGACCTACTTCTCATAC | TTGCCTCAGATAAGACCTC |
| *EsFLS* | GGATTGGACTTGAACCTAAC | CCTTGAACACTTGAAGACC |
| *EsFNS II* | GAATGGGCAATGTCAGAAC | TAGATGGCGTGGATGTATG |
| *EsDFR1* | CTGCTGGAACTGTTGATG | CTAGTGTTGGTATGATACTGATG |
| *EsDFR2* | TCCATCCGTTACTGTCAC | TTCACCTTCTTCATGTTAGC |
| *EsF3'H* | GCTTGGTGAGTGAGTCTG | TTCTTTGGGATGTGGTAAC |
| *EsF3'5'H* | ATCTGGAATGGTTGTTGC | TCTTTGGGACGGTTTGAG |
| *EsANS* | GTCAGATTCAAGGTTATGGTAG | CTTAGTCGTGTAGCATATTCG |
| *EsLAR* | TCATCGGTGGCTTCATTG | CTTGTCCTGTAGTTCTTTGTG |
| *EsRT* | TCATCAGCCTATCACTTAGTC | CTCCTTCCATAGCCGTAG |
| *EsUF3GT* | GGCTGTTAAGTTGGTGATG | GTTCTTGATTGAAGTTGTCTATG |
| *EsUF7GT* | GTGAGGGTGAAGGTGAATGAA | CTTTCCTGGACAACTCCTTCC |
| *EsACT1* | ATGAAAGTAATAGAGGAAAGCC | TGGATGAAACAGGAGGTAG |
| *EsTT8* | GTGGCTACTCAAGTACATTC | GATTCGCACTCAACTCATC |
| *EsGL3* | TGCTTGAAGAGGAGTGAG | CGTTGCCAGAGTTGAATG |
| *EsTTG1* | TACTTCCAGCATCGACAC | ATCCTAACAGAGCCATCAG |
| *EsMYBF1* | AATAGGTGGTCACTGATTGCTGC | CACCCATCTTGGCTAAGTTCATC |
| *EsMYBA1* | TGGTCGGACAGCCAATGATG | AGGGGGTAGTATCCTTTCCTTGTC |
| *EsMYB12* | CGAAGAGGGGTCATCTCCTAC | CCTTTGCCACGAATAAGATGT |
| *EsDET1* | ATACTGATCACCAGCCCTCCT | AGCATGGACGTTATTGGAGTG |
| *EsHY5* | TGTTGAGAAATCGGGTTTCAG | TCTTCCAATTTGGCATTCTTG |
| *EsActin* | TACGAACAGGAGCTGGAGACTT | GATGGTCCAGACTCGTCATACTC |

**Table S3. Correlation analysis of flavonoid-related genes regarding the gene expression profiles and the accumulation patterns of the four BCs in the HN3 and JX3 lines**

| **Gene** | **HN3** | **JX3** | **Gene** | **HN3** | **JX3** |
| --- | --- | --- | --- | --- | --- |
| *EsC3H* | 0.50^※※N^ | 0.31^※N^ | *EsCHS2* | NS | 0.70^※※P^ |
| *EsCHS3* | 0.36^※N^ | 0.72^※※N^ | *EsCHI1* | 0.27^※P^ | NS |
| *Es4CL1* | 0.46^※※N^ | 0.33^※N^ | *EsF3H* | NS | 0.46^※※P^ |
| *Es4CL2* | 0.27^※N^ | 0.67^※※N^ | *EsANS* | 0.47^※P^ | NS |
| *Es4CL3* | 0.25^※P^ | 0.53^※※P^ | *EsLAR* | NS | 0.51^※※N^ |
| *EsCHI2* | 0.46^※※N^ | 0.80^※※N^ | *EsUF3GT* | NS | 0.51^※※P^ |
| *EsDFR2* | 0.48^※※N^ | 0.39^※※N^ | *EsRT* | NS | 0.41^※※P^ |
| *EsUF7GT* | 0.44^※※N^ | 0.52^※※N^ | *EsCA3OMT* | NS | 0.27^※P^ |
| *EsMYB12* | 0.38^※N^ | 0.87^※※N^ | *EsGL3* | NS | 0.88^※※P^ |
| *EsTT8* | 0.42^※※N^ | 0.27^※N^ | *EsTTG1* | NS | 0.39^※P^ |
| *EsMYBF1* | 0.39^※※P^ | 0.76^※※P^ | *EsDET1* | NS | 0.26^※N^ |
| *EsMYBA1* | 0.55^※※P^ | 0.86^※※P^ | *EsHY5* | 0.25^※N^ | NS |

^※^, *P* ≤ 0.05; ^※※^, *P* ≤ 0.01; N, negative correlation; P, positive correlation; NS, not significant.

**Table S4. Correlation analysis of expression levels between *EsMYB12* and other structural genes involved in the phenylpropanoid pathway**

| **Gene** | **HN3** | **JX3** |
| --- | --- | --- |
| *EsC3H* | 0.52^※※P^ | 0.46^※※P^ |
| *EsC4H* | NS | NS |
| *Es4CL1* | 0.81^※※P^ | 0.50^※※P^ |
| *Es4CL2* | NS | 0.73^※※P^ |
| *EsCHI1* | 0.56^※※N^ | NS |
| *EsCHI2* | 0.67^※※P^ | 0.71^※※P^ |
| *EsCHS1* | NS | NS |
| *EsCHS2* | NS | 0.71^※※N^ |
| *EsCHS3* | NS | 0.87^※※P^ |
| *EsFNS II* | NS | NS |
| *EsF3'H* | NS | NS |
| *EsF3'5'H* | NS | 0.24^※N^ |
| *EsF3H* | NS | 0.47^※※N^ |
| *EsDFR1* | NS | NS |
| *EsDFR2* | 0.27^※P^ | 0.51^※※P^ |
| *EsLAR* | NS | 0.43^※※P^ |
| *EsFLS* | 0.22^※N^ | NS |
| *EsANS* | 0.64^※※N^ | NS |
| *EsUF7GT* | 0.42^※※P^ | 0.61^※※P^ |
| *EsACT1* | NS | NS |

^※^, *P* ≤ 0.05; ^※※^, *P* ≤ 0.01; N, negative correlation; P, positive correlation; NS, not significant.

**Table S5. List of primer sequences for the qPCR assay in tobacco**

| Gene name | Accession number | Forward primer (5’-3’) | Reverse primer (5’-3’) | PCR length |
| --- | --- | --- | --- | --- |
| *NtPAL* | X78269 | CGATAGACTTGAGGCATTTG | AGGTTCTCTTAGCGACTTG | 78 |
| *NtC4H* | AB236952 | GTTGCCTCCTCCAGGACAGTC | TGGTGGAATGCTTCAAAATGTG | 83 |
| *Nt4CL* | U50845 | GCGACATTGGGTTCATTG | TTCTCCTGCTTGCTCATC | 176 |
| *NtCHS* | AF311783 | AGCGAGCATAAGGTTGAG | ACCACCACTATGTCTTGTC | 164 |
| *NtCHI* | AB213651 | CTTTTCTCGCCGCTAAATG | TTTCTGCCACCTTCTCTG | 159 |
| *NtF3H* | AB289450 | GAGGCAATGGGCTTAGAG | TCAGTGTGTCGTTTCAGTC | 128 |
| *NtF3'H* | AB289449 | AGCCATAGTCAAGGAAACC | CTCACAACTCTCGGATGC | 79 |
| *NtDFR* | EF421429 | TAAGAAGATGACAGGATGGATG | TGGCGGTATGATGCTAATG | 109 |
| *NtFLS* | DQ435530 | GTCCCATATAACCATTCTTGTC | CACTCTTGTATTTCCCATTGC | 152 |
| *NtANS* | AB289447 | CTACATTCCAGCAACAAGTG | GTCCCAGCCCAATAGAAAG | 86 |
| *NtTub1* | AJ421411 | TCCGTGGTGATGTTGTG | TGGTGGCTGATAGTTGATAC | 125 |
