# Supplementary figures and images for: Elucidating the biosynthetic and regulatory mechanisms of flavonoid-derived bioactive components in Epimedium sagittatum
Source: Front Plant Sci. 2015 Sep 3;6:689. doi: 10.3389/fpls.2015.00689 (PMC4558469; doi:10.3389/fpls.2015.00689)

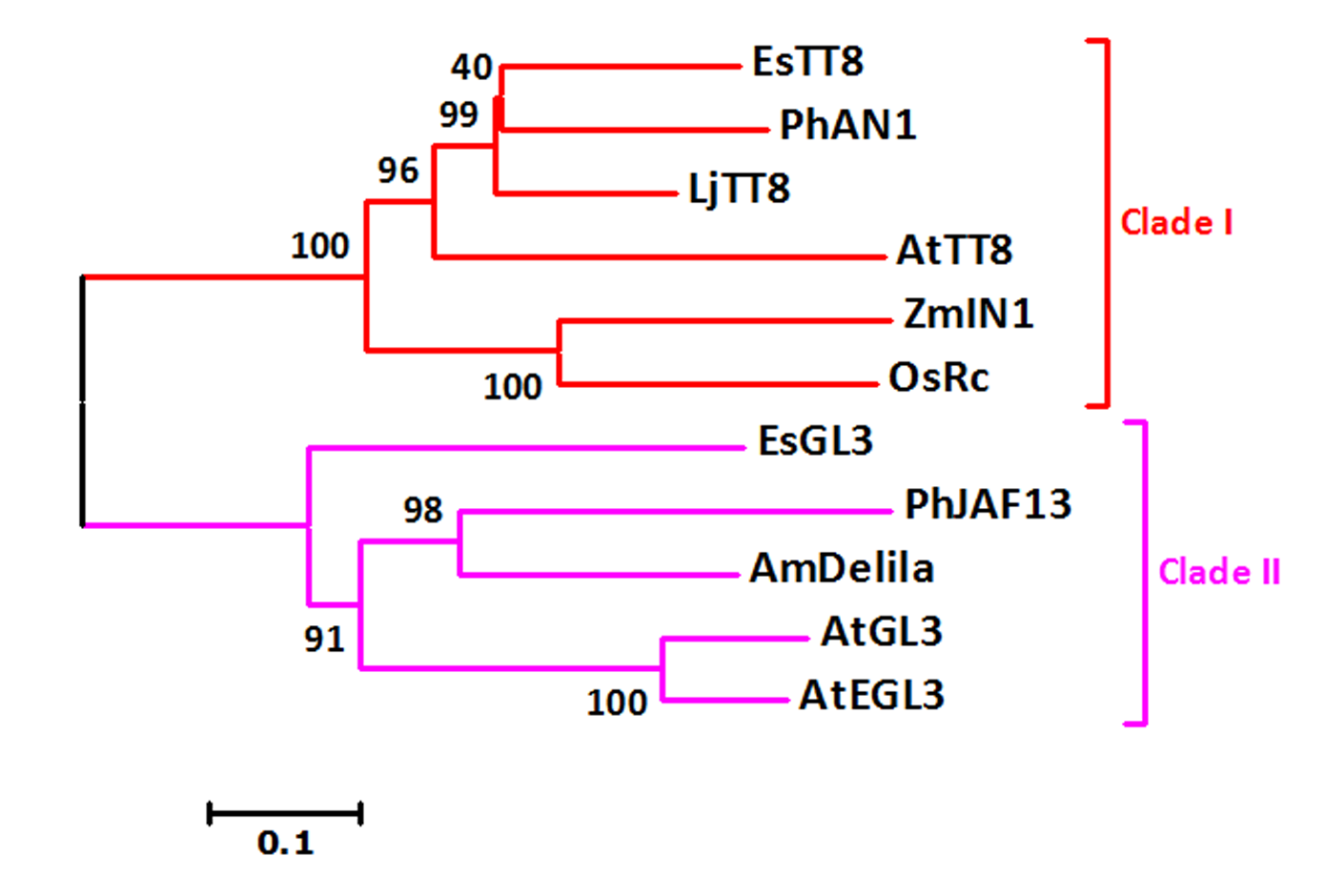

Supplement: Figure S1 — Phylogenetic relationship analysis of two bHLH transcription factors, EsTT8 and EsGL3, from E. sagittatum and other plant bHLH proteins. This phylogenetic tree was constructed using the neighbor-joining method by the MEGA 5 software. The scale bar represents the number of substitution per site and the numbers next to the nodes are bootstrap values from 1000 replicates. All bHLH protein sequences were retrieved from the GenBank database and accession numbers are as follows: Epimedium sagittatum, EsTT8 (AGT39063); Petunia x hybrida, PhAN1 (AAG25927), PhJAF13 (AAC39455); Lotus japonicus, LjTT8 (BAH28881); Arabidopsis thaliana, AtTT8 (CAC14865), AtGL3 (NP_680372), AtEGL3 (NP_176552); Zea mays, ZmIN1 (AAB03841); Oryza sativa, OsRc (BAF42668); Antirrhinum majus, AmDelila (AAA32663). [file Image1.TIF]

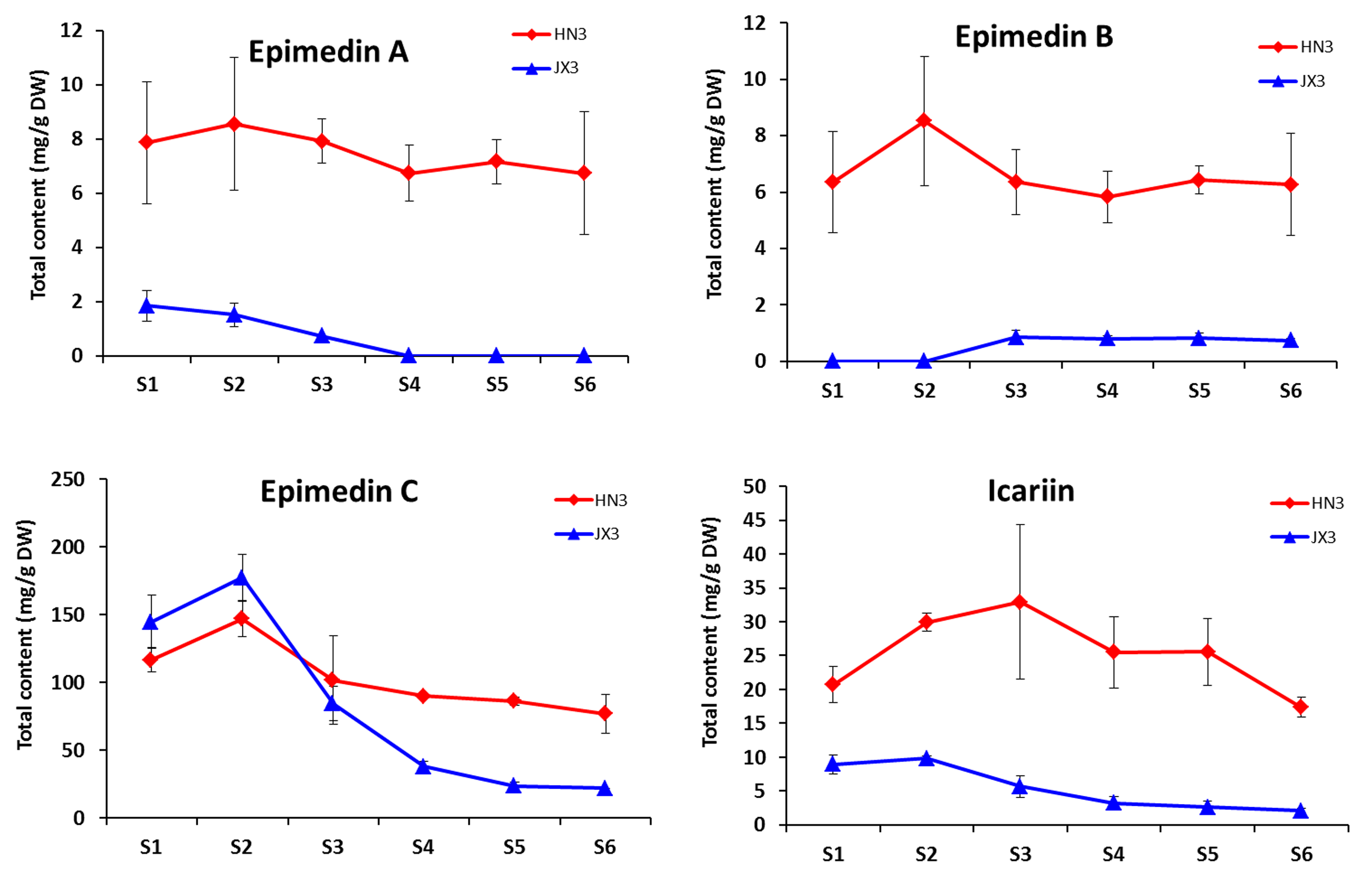

Supplement: Figure S2 — The accumulation patterns of epimedin A, epimedin B, epimedin C, and icariin in the HN3 and JX3 lines of E. sagittatum during the leaf developmental stages. Each data represents the mean value plus SD (standard deviation) from three biological replicates. [file Image2.TIF]

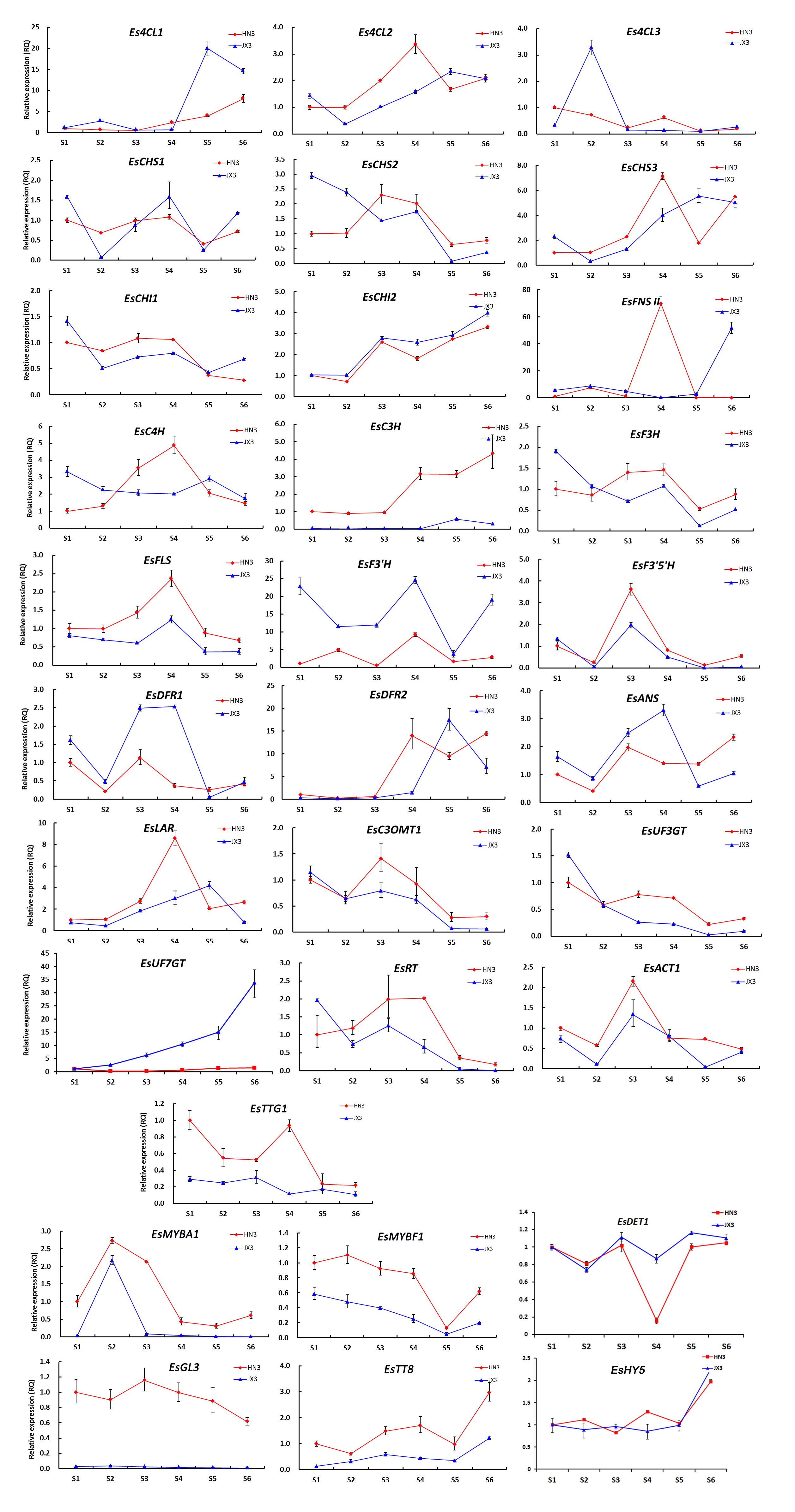

Supplement: Figure S3 — The expression profiles of flavonoid-related genes in the HN3 and JX3 lines of E. sagittatum during the six developmental stages of leaves. The comparative Ct method was used to determine the relative expression level. Each date represents the mean value plus SD (standard deviation) error from three technical replicates. [file Image3.TIF]

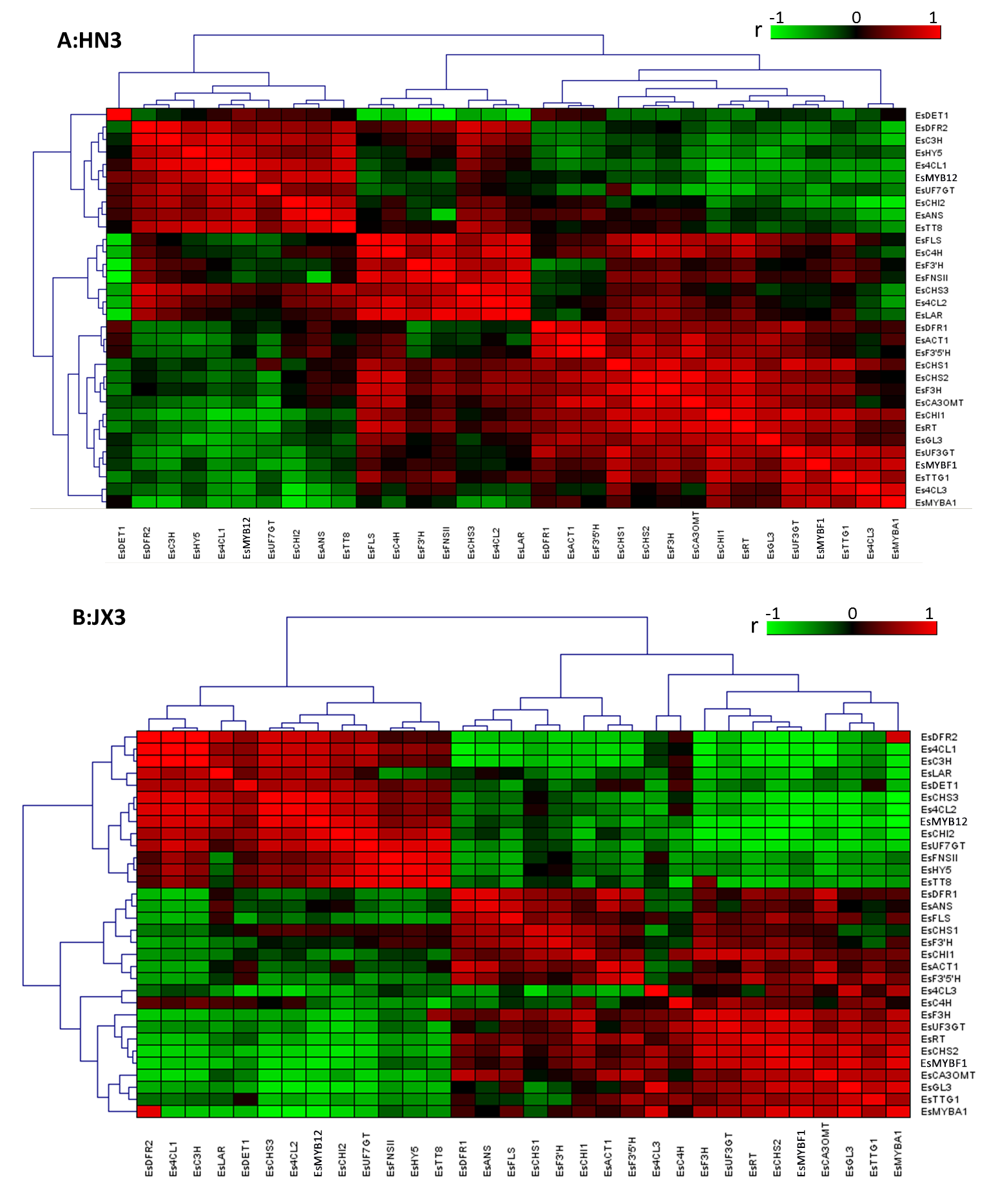

Supplement: Figure S4 — Coexpression analysis of 32 candidate genes involved in the phenylpropanoid/flavonoid pathway in the HN3 (A) and JX3 (B) lines of E. sagittatum based on the Genesis method. The correlation coefficient R is indicated by the color bar at upper right corner. [file Image4.TIF]

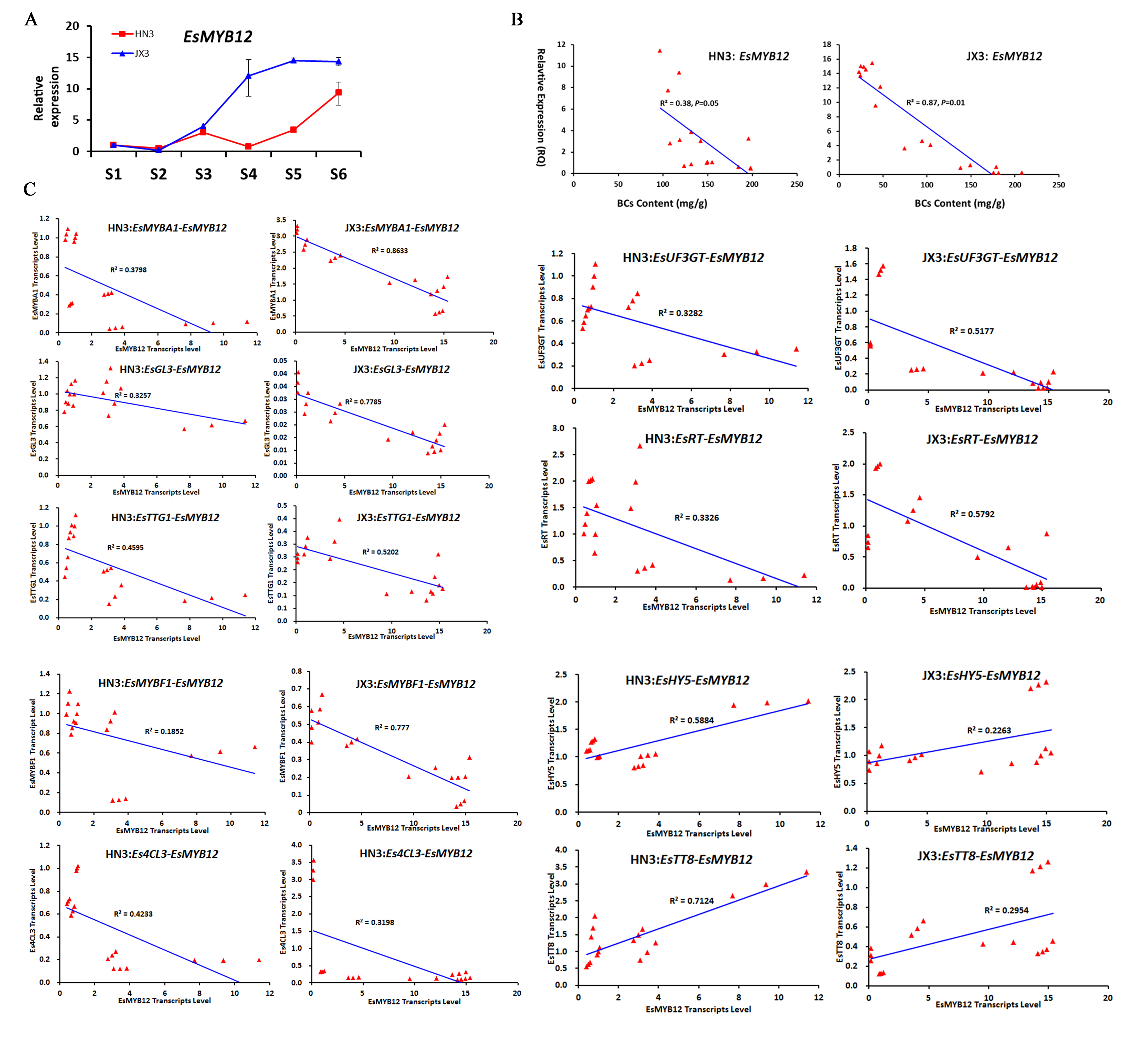

Supplement: Figure S5 — The expression profile (A) and correlation analysis of EsMYB12 in relation to the four BC accumulation patterns (B) with other flavonoid-related genes (C) during the developmental stages of E. sagittatum leaves. [file Image5.TIF]

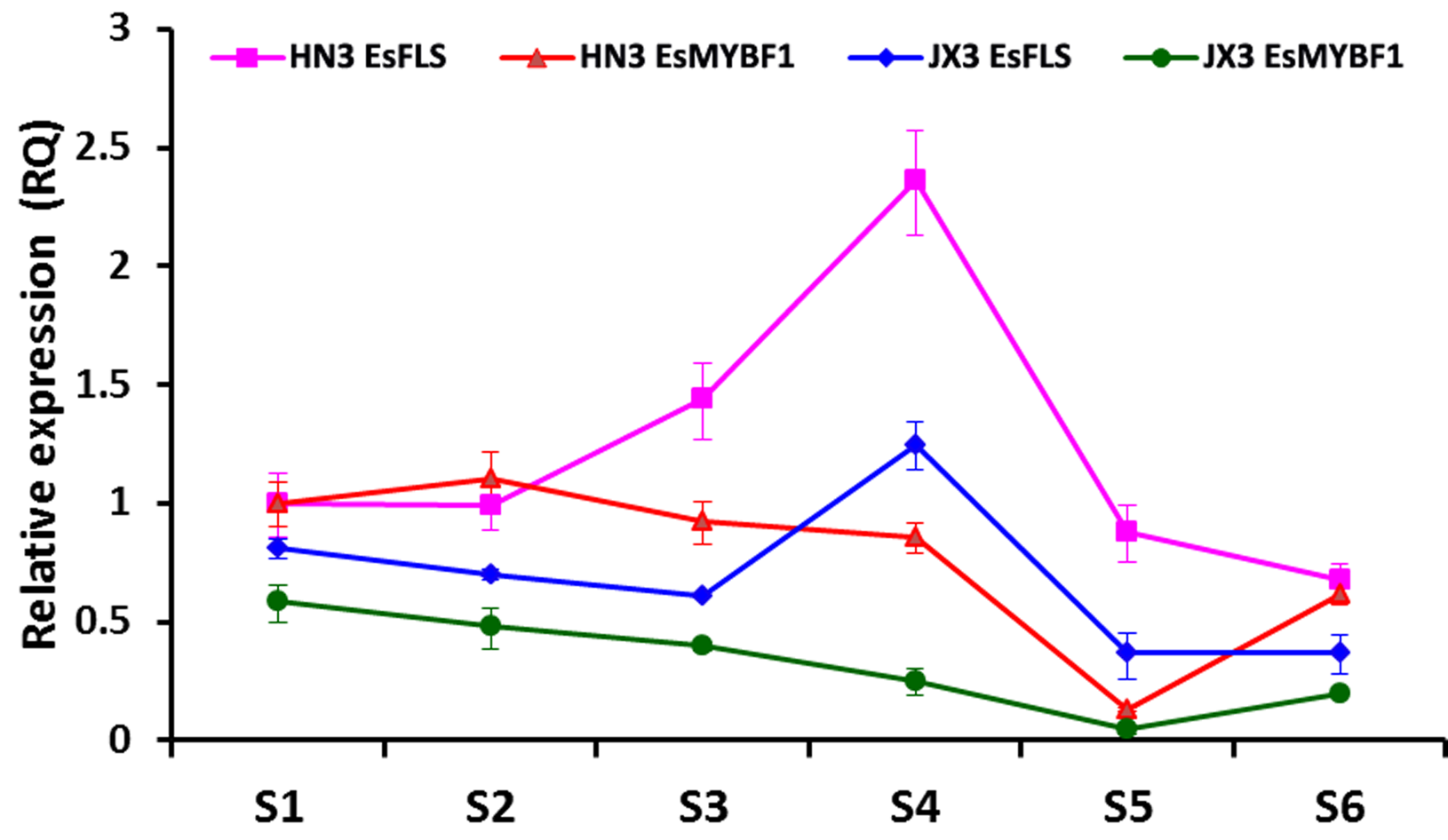

Supplement: Figure S6 — The expression patterns of the EsMYBF1 and EsFLS genes in the HN3 and JX3 lines of E. sagittatum during the developmental stages of leaves. [file Image6.TIF]
